# Left ventricular ejection fraction using a simplified wall motion score based on mid-parasternal short axis and apical four-chamber views for non-cardiologists

**DOI:** 10.1186/s12872-023-03141-x

**Published:** 2023-03-08

**Authors:** Réal Lebeau, Maxime Robert-Halabi, Maxime Pichette, Alain Vinet, Claude Sauvé, Maria Dilorenzo, Viet Le, Eric Piette, Mathieu Brunet, William Bédard, Karim Serri, Frédéric Poulin

**Affiliations:** 1 Division of Cardiology, Hôpital du Sacré-Coeur de Montréal, 5400 Gouin Blvd W., Montreal, QC H4J 1C5 Canada; 2 Centre de Rercherche de l’Hôpital Sacré-Coeur de Montréal, Montreal, QC Canada; 3 Department of Pharmacology and Physiology, Faculty of Medicine, Université de Montréal, Montreal, QC Canada; 4 Department of Emergency Medicine, Hôpital Sacré-Coeur de Montréal, Montreal, QC Canada

**Keywords:** Left ventricular ejection fraction, Wall motion score, POCUS

## Abstract

**Background:**

There is a need for a convenient, yet reliable method to assess left ventricular ejection fraction (LVEF) with point-of-care ultrasound study (POCUS). We aim to validate a novel and simplified wall motion score LVEF based on the analysis of a simplified combination of echocardiographic views.

**Methods:**

In this retrospective study, transthoracic echocardiograms of randomly selected patients were analysed by the standard 16-segments wall motion score index (WMSI) to derive the reference semi-quantitative LVEF. To develop our semi-quantitative simplified-views method, a limited combination of imaging views and only 4 segments per view were tested: (1) A combination of the three parasternal short-axis views (PSAX BASE, MID-, APEX); (2) A combination of the three apical views (apical 2-chamber, 3-chamber and 4-chamber) and (3) A more limited combination of PSAX-MID and apical 4-chamber is called the MID-4CH. Global LVEF is obtained by averaging segmental EF based on contractility (normal = 60%, hypokinesia = 40%, and akinesia = 10%). Accuracy of the novel semi-quantitative simplified-views WMS method compared to the reference WMSI was evaluated using Bland–Altman analysis and correlation was assessed in both emergency physicians and cardiologists.

**Results:**

In the 46 patients using the 16 segments WMSI method, the mean LVEF was 34 ± 10%. Among the three combinations of the two or three imaging views analysed, the MID-4CH had the best correlation with the reference method (r^2^ = 0.90) with very good agreement (mean LVEF bias = − 0.2%) and precision (± 3.3%).

**Conclusions:**

Cardiac POCUS by emergency physicians and other non-cardiologists is a decisive therapeutic and prognostic tool. A simplified semi-quantitative WMS method to assess LVEF using the easiest technically achievable combination of mid-parasternal and apical four-chamber views provides a good approximative estimate for both non-cardiologist emergency physicians and cardiologists.

**Supplementary Information:**

The online version contains supplementary material available at 10.1186/s12872-023-03141-x.

## Background

A new era is emerging in echocardiography with point-of-care ultrasound study (POCUS). One of the challenges of POCUS is to reliably assess left ventricular (LV) ejection fraction (LVEF). Cardiologists estimate LVEF visually, semi-quantitatively using the wall motion score (WMS) or quantitatively using the modified Simpson’s biplane method of disks. However, for less experienced physicians using POCUS, methods currently taught (M-Mode LV fractional shortening, E point septal separation) are oversimplifications that yield suboptimal estimates of systolic function in a significant proportion of cases [1]. To the untrained eye, a qualitative rapid visual estimation of the LVEF can be challenging with the limited views available in POCUS [2]. There is a need for a convenient and reliable method to assess LVEF with POCUS.

Our study aims to validate a novel simplified semiquantitative method to estimate LVEF based on regional wall motion analysis using a limited combination of views.

## Methods

Forty-six non-consecutive cases with a wide range of LVEF values and sufficient quality of their transthoracic echocardiographic images were randomly selected from an educational database gathered over the years (2018–2020, n = 31; 2013–2017, n = 9; 2009–2012. n = 6). This database was created to provide teaching examples to residents and fellows learning the wall motion score analysis. Cases were selected in a non-biased fashion to provide a random representative sample of echocardiography cases seen routinely by clinicians. Patients with poor diagnostic quality of echocardiographic images or LVEF greater than 65% (global hyperkinesis) were excluded. Furthermore, patients with severe valvular disease, hypertrophic cardiomyopathy, or congenital heart disease were also excluded. The study was approved by our institution Research Ethics Board and written informed consent was waived.

### Reference LVEF value

An experienced cardiologist (RL) determined the 16-segments left ventricular WMS index (WMSI), which implies the full analysis of LV contractility using the 6 standard views (parasternal base, mid and apical and apical 4-, 3-, and 2-chamber planes) (Additional file 1: Figure S1). Based on the American Society of Echocardiography score, LVEF was derived: LVEF = 90–26 × WMSI (Additional file 2: Table S1) [3]. This regression equation has been validated in previous echocardiographic and cardiac magnetic resonance (CMR) imaging studies [4–6] and was used as our reference LVEF value.

### Validation of a Novel semi-quantitative simplified-views WMS-EF method

To develop our semi-quantitative simplified-views method, the selected patient images were re-analyzed, in a blinded fashion, using a limited combination of imaging views and only 4 segments per view (as opposed to 6 with the reference 16-segments method). Three combinations were tested in all patients:

1. A combination of the three parasternal short-axis views (PSAX). (Mitral level base (PSAX-B), papillary muscle level (PSAX-MID) and apex (PSAX-A)) called the PSAX-BMA.
2. A combination of the three apical views (AP). (2-chamber, 3-chamber and 4-chamber), called the 234CH.
3. A more limited combination of PSAX-MID and apical 4-chamber, called the MID-4CH.

**Fig. 1:**
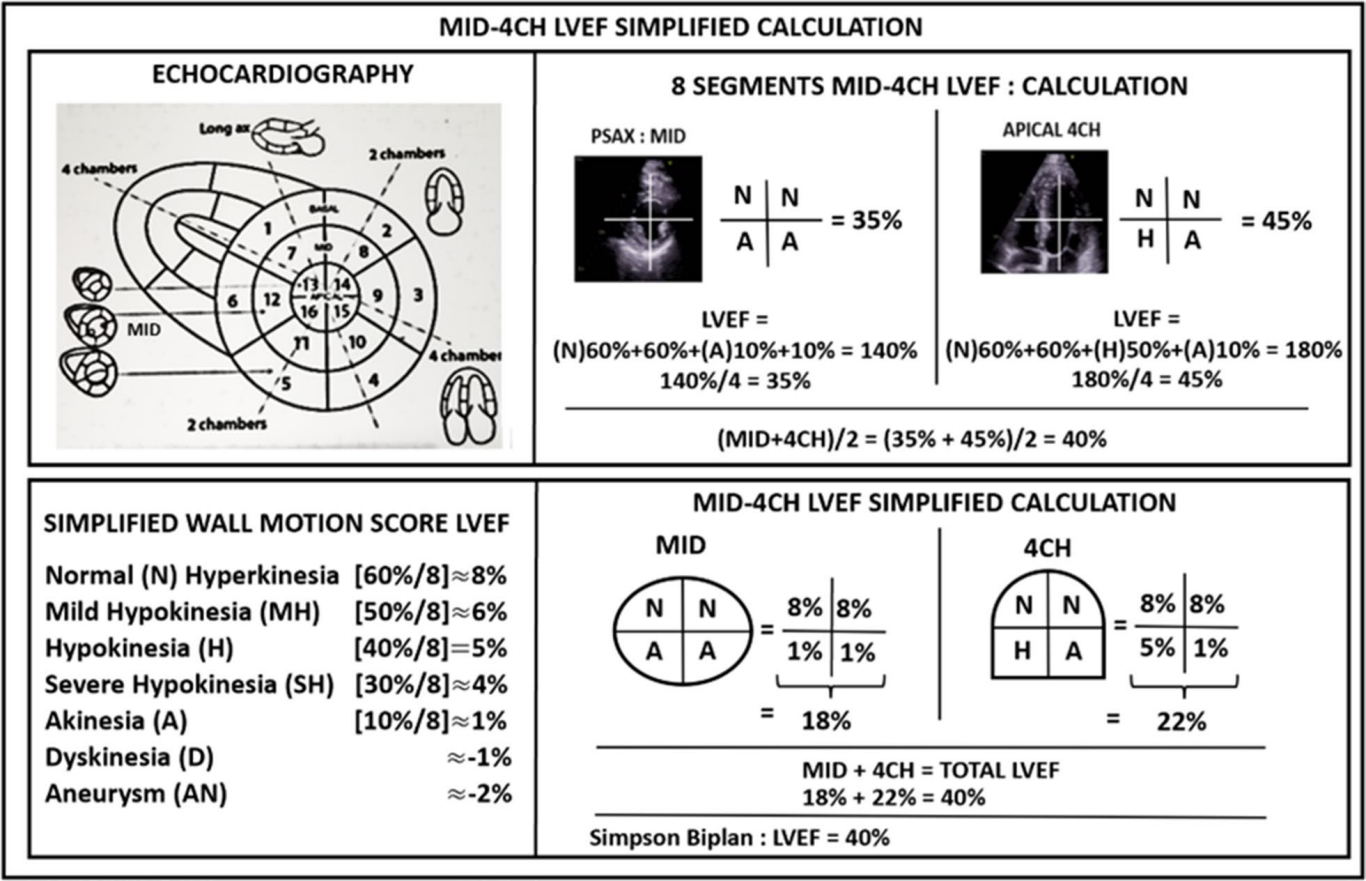
The MID-4CH simplified WMS-EF method. To calculate the simplified LVEF, the average of all 8 segments LVEF was obtained. Segment LVEF = Normal (N) = 60%; mild hypokinesia = 50%; hypokinesia (H) = 40%; severe hypokinesia = 30%; akinesia (A) = 10%; dyskinesia = − 10%; aneurysm = − 20%. An alternative easier and practical way to calculate the global LVEF is also shown with this case example. In this patient with involvement of the circumflex and the right coronary artery, the MID-4CH simplified WMS LVEF was 40% versus 40% by the Simpson’s biplane EF method

With the novel simplified-views WMS method, each view was divided into 4 segments. For each prespecified segments, the movement and thickening of the myocardium were analyzed (Fig. 1). We derived a simplified score from our previous echo and CMR studies (Additional file 2: Table S1). If the overall movement and thickening of the segment were normal, the LVEF for that specific segment was 60%, and 10% if it was akinetic (no thickening and no movement). If the thickening and mobility were moderately impaired, the segment was labelled as hypokinetic and the LVEF was 40%. Mild or severe hypokinesia corresponded to segmental LVEF values of 50% and 30%, respectively. Finally, dyskinesia (outward systolic movement of a segment) and aneurysm (systolo-diastolic deformation) corresponded to segmental LVEFs of -10% and -20%, respectively. In this simplified score, localized hyperkinesia was considered equivalent to normokinesia. To calculate the overall LVEF, the average of each segment LVEF was obtained (Fig. 1, Additional file 3: Videos 1 Additional file 4: Video 2, Additional file 5: Videos  3). The novel semi-quantitative simplified-views WMS-EF method and the 16-segment wall motion score index were compared. In addition, LVEF derived using the novel semi-quantitative simplified-views WMS-EF method was compared with the reference biplane Simpson’s method of disks (n = 40, 6 patients excluded due to inadequate endocardial definition precluding precise diastolic or systolic tracings).

### Exploration of the novel semi-quantitative simplified-views WMS method for LVEF assessment among emergency physicians

In order to test the validity of our novel semi-quantitative simplified-views WMS method, 3 emergency physicians with training in cardiac POCUS and 5 echocardiographers used the semi-quantitative simplified-views WMS method in 10 patients from the initial cohort. Systolic function ranged from low LVEF (≤ 30%): 15%, 19%, 19% and 22%; moderate or intermediate LVEF (31–49%): 40%, 43% and 43% as well as normal LVEF (≥ 50%): 53%, 64% and 64%.

### Statistical analysis

Normality of distribution of continuous variables was assessed with the Shapiro–Wilk test. Continuous variables were described as mean ± standard deviation (SD) if the distribution was normal, otherwise as median and interquartile range (IQR) (25th–75th). The novel semi-quantitative simplified-views WMS-EF method and the 16-segment wall motion score index were compared by linear regression analysis and Bland–Altman analysis. Correlation was assessed by the Pearson coefficient of determination (r^2^) using the bootstrap resampling method (1000 samples) to determine the 95% confidence intervals (CI) (bias-corrected and accelerated method). Bootstrapping is an efficient approach to assess average model performance (internal validation) in small cohorts. Intra- and inter-observer variabilities were evaluated with intraclass correlation coefficients. All statistical analyses were conducted using SPSS (IBM SPSS Statistics for Windows, Version 25.0. Armonk, NY: IBM Corp.).

## Results

Diagnostic quality data were obtained in 46 subjects (mean age 66, range from 48 to 88 yrs, 22% female) with mean LVEF of 34 ± 10% (LVEF values ranging from 15 to 56%). The patients correspond to an unbiased random sample with no specific criteria and represent various unselected clinical indications encountered in our large teaching hospital (assessment of non-ischemic (26%) or ischemic (59%) cardiomyopathies, arrhythmias (5%), and other diagnoses (10%)).

### Reference WMS-LVEF method

The average WMS using the standard 16-segments method in 6 imaging planes was 34 ± 6 (range 21–46 corresponding to a WMSI = 2.1 ± 0.4 (range 1.3–2.9) or to a mean WMSI-LVEF of 34 ± 10%. The distribution of LVEF was as follows LVEF ≥ 50%, n = 12 (25%); LVEF 31–49%, n = 19 (42%); LVEF ≤ 30%, n = 15 (33%). Segments with localized aneurysm (9 patients) or dyskinesia (n = 5) were represented.

### Our proposed semi-quantitative simplified-views WMS-EF method

Among the three combinations of the two or three imaging views analysed, the MID-4CH had the best correlation with the reference 16 segments WMSI method (n = 46; r^2^ = 0.90, p˂0.001; bootstrap 95% CI spans from 0.85 to 0.95).

### Analysis of systematic bias

Bland–Altman analysis showed good agreement between the MID-4CH simplified WMS method and the WMSI-LVEF (mean LVEF bias = -0.2%). The SDs of the distribution of inter-method differences were acceptable (± 3.3%) (Additional file 6: Figure S2).

### Exploration of the novel semi-quantitative simplified-views method for LVEF assessment among emergency physicians

The MID-4CH simplified WMS analysis of 10 patients with a wide range of LVEF (38 ± 19%; range 15–64%) by the 3 emergency physicians (r^2^ = 0.90) and 5 cardiologists (r^2^ = 0.84) also correlated strongly with the reference standard (Additional file 7: Table S2).

Results of the other combinations of imaging (PSAX-BMA and Apical 234CH) are also presented (Additional file 7: Table S2).

### Simpson biplane analysis

LVEF of 40 patients using Simpson’s biplane method of disks was measured (37.5 ± 13%) and compared well with our MID-4CH simplified WMS-EF estimation (r^2^ = 0.92, p˂0.001; bootstrap 95% CI spans from 0.88 to 0.96). Bland–Altman analysis showed good agreement between the MID-4CH simplified WMS method and the Simpson-LVEF (mean LVEF bias; new score versus Simpson’s = − 2.2%). The SDs of the distribution of inter-method differences were acceptable (± 5.3%). Similar correlation with was obtained among 5 cardiologists (n = 10; r^2^ = 0.92) and 3 emergency physicians (n = 10; r^2^ = 0.91) performing the proposed MID-4CH analysis.

### Reproducibility

Intra and inter-observer variations of our semi-quantitative MID-4CH WMS-EF results using data from our reference echocardiographer and all 5 echocardiographers in 10 randomly selected patients demonstrated good agreement between observations (intra-observer intraclass correlation coefficient = 0.95; inter-observer intraclass correlation coefficient = 0.92).

## Discussion

### The MID-4CH simplified method

The combination of two easily obtainable echocardiographic views (PSAX-MID and AP-4CH) using our WMS system is an accurate and rapid tool for LVEF estimation and correlated strongly with the 16-segments WMSI-derived LVEF and the Simpson’s biplane method. Results were reliable whether cardiologists or emergency physicians were reviewing the images.

PSAX-MID and AP-4CH are views frequently used in POCUS cardiac ultrasound imaging [2]. Using a simplified wall motion score based on these 2 views is a reasonable assumption since they include myocardial segments perfused by each of the 3 coronary vessels, which would prevent missing a large infarct in one of these territories (Additional file 8: Figure S3). Indeed, we found that the MID-4CH WMS-EF method was reliable in patients with myocardial infarctions whether it was an inferior (n = 13; r^2^ = 0.93), or an anterior infarct (n = 13; r^2^ = 0.86), or triple-vessels disease (n = 15; r^2^ = 0.81). Also, the apical 4-chamber view is of particular interest since it is easier to observe dyskinesia or aneurysm compared to the short axis views.

### The use of the semiquantitative wall motion score

The validity of semi-quantitative 16-segments LV wall motion scores has previously been established in a systematic review [7] and also by our group [4, 6] using isotopic ventriculography and CMR as a reference value. We have also shown that the assessment of wall motion abnormalities by novice readers in echocardiography was better than a global visual estimation of LVEF [8]. We believe that the results of the current analysis will further simplify LVEF assessment in POCUS since only two views (PSAX-MID and AP-4CH) are required to obtain a reliable LVEF estimate.

### LVEF assessment by non-cardiologists

Previous studies have compared assessment methods of LVEF by non-cardiologists with minimal training and have shown that cardiac POCUS is a useful tool. However, agreement rate in LVEF estimation varied widely [9, 10]. In a recent study involving 113 patients at a large academic tertiary-care center, quantitative measurements of E point septal separation and fractional shortening demonstrated poor accuracy in estimating of LVEF (r = 0.70 and 0.85, respectively), even among experienced sonographers [1]. Visual estimation performed better but remains subjective and experience-dependant [11]. Our novel method which is semi-quantitative, more objective and less expert-dependant provides a rapid and accurate assessment of LVEF using only two key echocardiographic views (PSAX-MID and AP-4CH). Contrary to E point septal separation and visual estimation, which provide interval % estimate of systolic function, the novel MID-4CH simplified wall motion method giving a precise point % estimate of LVEF with minimal bias and good precision considering the intrinsic limitations of any POCUS cardiac examination.

### Limitations

The external validity of our study is limited by the fact than we excluded patients with poor image quality. Left ventricle with unusual morphology, such as complex congenital heart disease, severe asymmetric wall thickening, or hyperkinesia may not be adequately assessed by the MID-4CH simplified LVEF method. Moreover, although user-friendly, our semi-quantitative method requires a minimal training in cardiac POCUS to interpret regional WMA [12]. Also, our reference values were not directly obtained from nuclear medicine or CMR, but from a validated WMSI-derived formula [4]. Finally, improving endocardial definition in TTE with the use of contrast agents could have resulted in improved precision of the new simplified wall motion score based on mid-parasternal short axis and apical four-chamber views.

## Conclusion

Cardiac POCUS by emergency physicians and other non-cardiologists is a decisive diagnostic and prognostic tool. We have shown that a novel simplified semi-quantitative WMS method to assess LVEF using the easiest technically achievable combination of mid-parasternal and apical four-chamber views provide a good approximative estimate by both non-cardiologist emergency physicians and cardiologists.

## Supplementary Information

**Additional file 1**.** Figure 1**. LVEF by WMSI using the reference 16-segments method. Each segment is given a score based on its systolic function (normal N = 1, hypokinesis H = 2, akinesis A = 3). The index (WMSI) is calculated by dividing the total of the wall motion scores of each segment by 16. This patient with an anterior myocardial infarction had a wall motion score of 34 (WMSI = 2.13 (34/16)) which corresponds to a LVEF = 35%.Legend: A = akinetic, H: hypokinetic, N: normal, Ant: anterior, Ant-lat: antero-Lateral, Inf-Lat: infero-lateral, Inf: inferior, Lat: lateral, LV: Left ventricle, LVEF: Left ventricular ejection fraction, RV: right ventricle, WMSI: Wall motion score index.

**Additional file 2**.** Table 1**. The conversion of ECHO and CMR WMSI into LVEF by regression models in 3 studies

**Additional file 3**. **Video 1**. Complements the patient example from Fig. 1. Standard echocardiographic views to obtain the LVEF with the simplified MID-4CH method (Parasternal short-axis MID view is shown).

**Additional file 4**.** Video 2**. Complements the patient example from Fig. 1. Standard echocardiographic views to obtain the LVEF with the simplified MID-4CH method (Apical 4-chambers view is shown) and the reference biplane Simpson’s method  (Apical 4-chambers view is shown).

**Additional file 5**.** Video 3**. Complements the patient example from Fig. 1. Standard echocardiographic views to obtain the LVEF with reference biplane Simpson’s method (Apical 2-chambers view is shown).

**Additional file 6**.** Figure 2**. Comparison between LVEF by the 16-segments WMSI reference method and the MID-4CH simplified WMS method. Legend: WMSI, wall motion score index; LVEF, left ventricular ejection fraction; SD, standard deviation.

**Additional file 7**.** Table 2**. Correlation between a novel wall motion score method using different simplified combinations of views with the reference standard to estimate left ventricular ejection fraction

**Additional file 8**.** Figure 3**. Coronary circulation in the 6 echo views. The 4 apical segments are generally supplied from LAD coronary artery, but occasionally, the inferior part of the apex can be supplied by the right coronary artery and the lateral part by the circumflex coronary artery [3]. Legend: LAD, left anterior descending coronary artery; R, right coronary artery; Cx, Circumflex coronary artery

## Data Availability

Dr Frederic Poulin (f.poulin@umontreal.ca) should be contacted if someone wants to request the data.

## Abbreviations

234CH: A combination of the three apical views (2-chamber, 3-chamber and 4-chamber)
4CH: Apical 4-chamber view
AP: Apical views
CI: Confidence intervals
CMR: Cardiac magnetic resonance
IQR: Interquartile range
LVEF: Left ventricular ejection fraction
MID-4CH: A combination of the parasternal short-axis papillary muscle level and the apical 4-chamber views
POCUS: Point-of-care ultrasound study
PSAX: Parasternal short-axis
PSAX-A: Parasternal short-axis apical level
PSAX-B: Parasternal short-axis base level
PSAX-MID: Parasternal short-axis papillary muscle level
PSAX-BMA: A combination of the three apical views
R^2^: Coefficient of determination
SD: Standard deviation
WMS: Wall motion score
WMSI: Wall motion score index
