# Supplementary figures and images for: Left ventricular ejection fraction using a simplified wall motion score based on mid-parasternal short axis and apical four-chamber views for non-cardiologists
Source: BMC Cardiovasc Disord. 2023 Mar 8;23:115. doi: 10.1186/s12872-023-03141-x (PMC9993504; doi:10.1186/s12872-023-03141-x)

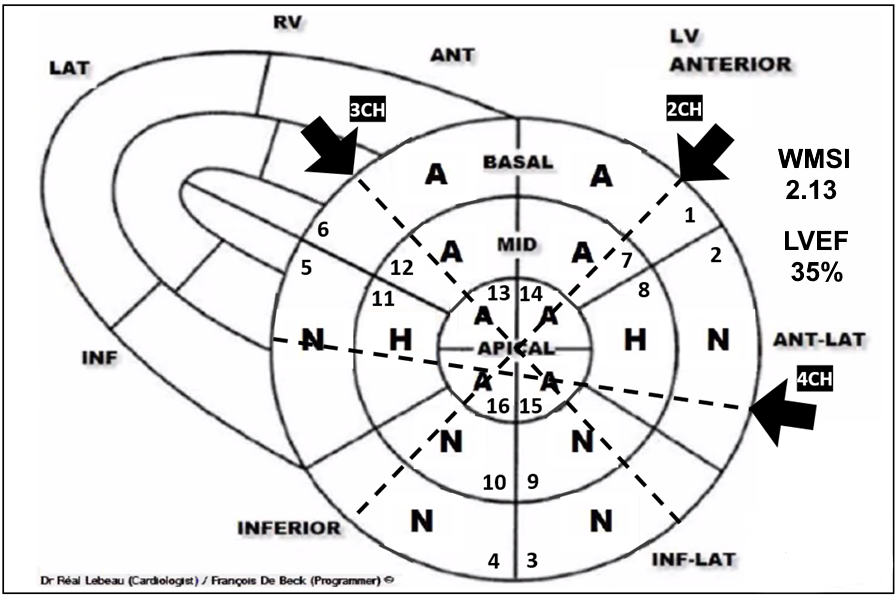

Supplement: Supplementary file 1 — Additional file 1. Figure 1. LVEF by WMSI using the reference 16-segments method. Each segment is given a score based on its systolic function (normal N = 1, hypokinesis H = 2, akinesis A = 3). The index (WMSI) is calculated by dividing the total of the wall motion scores of each segment by 16. This patient with an anterior myocardial infarction had a wall motion score of 34 (WMSI = 2.13 (34/16)) which corresponds to a LVEF = 35%.Legend: A = akinetic, H: hypokinetic, N: normal, Ant: anterior, Ant-lat: antero-Lateral, Inf-Lat: infero-lateral, Inf: inferior, Lat: lateral, LV: Left ventricle, LVEF: Left ventricular ejection fraction, RV: right ventricle, WMSI: Wall motion score index. [file 12872_2023_3141_MOESM1_ESM.tiff]

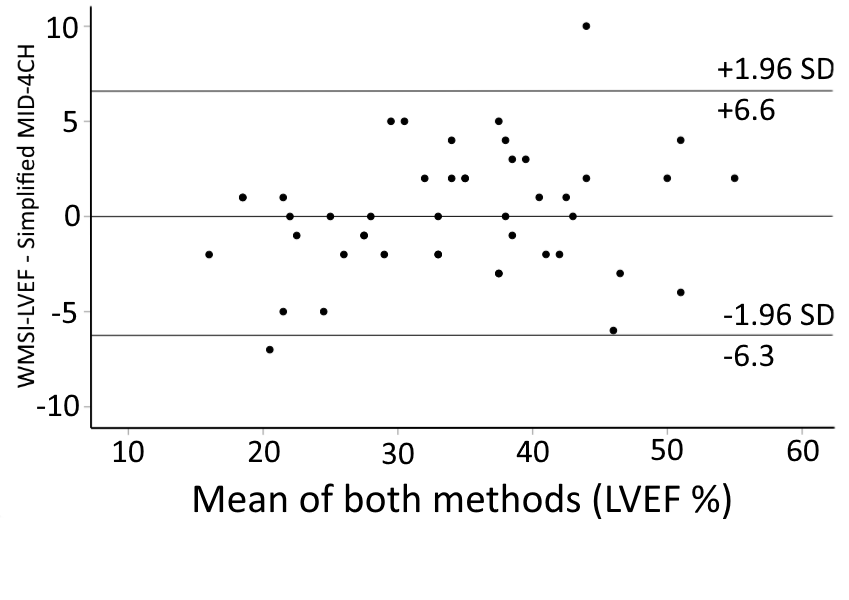

Supplement: Supplementary file 6 — Additional file 6. Figure 2. Comparison between LVEF by the 16-segments WMSI reference method and the MID-4CH simplified WMS method. Legend: WMSI, wall motion score index; LVEF, left ventricular ejection fraction; SD, standard deviation. [file 12872_2023_3141_MOESM6_ESM.tiff]

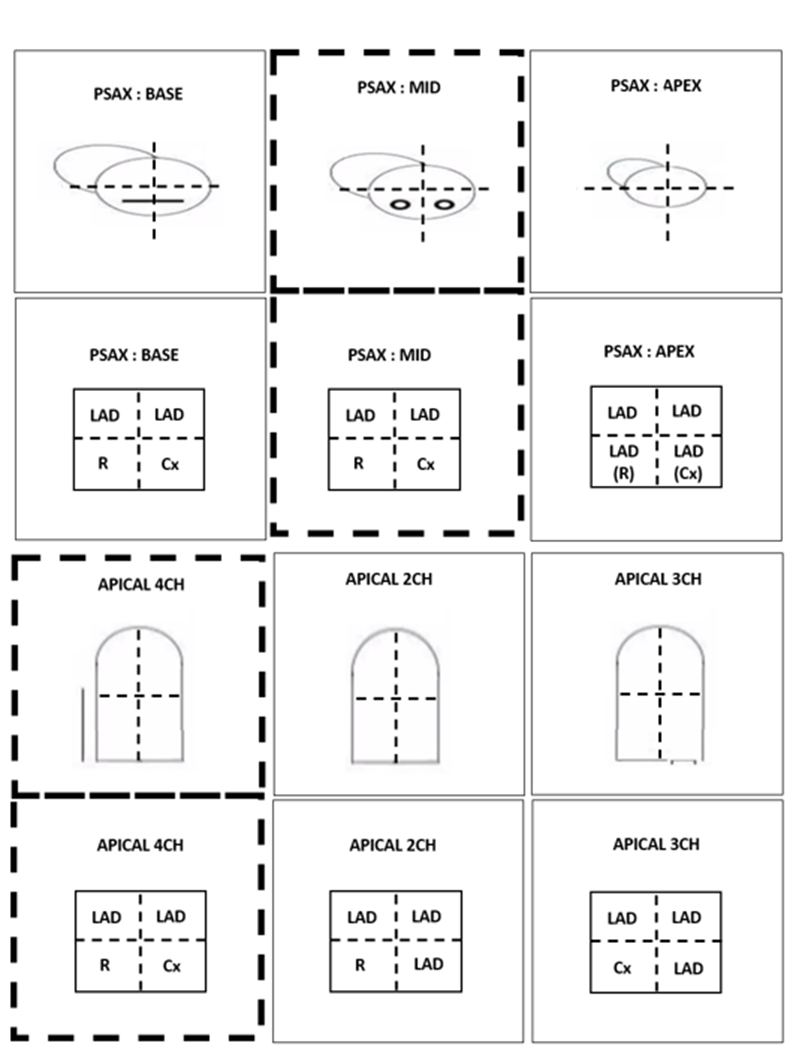

Supplement: Supplementary file 8 — Additional file 8. Figure 3. Coronary circulation in the 6 echo views. The 4 apical segments are generally supplied from LAD coronary artery, but occasionally, the inferior part of the apex can be supplied by the right coronary artery and the lateral part by the circumflex coronary artery [3]. Legend: LAD, left anterior descending coronary artery; R, right coronary artery; Cx, Circumflex coronary artery [file 12872_2023_3141_MOESM8_ESM.tiff]
