# Supplementary material for: Left ventricular ejection fraction using a simplified wall motion score based on mid-parasternal short axis and apical four-chamber views for non-cardiologists
Source: BMC Cardiovasc Disord. 2023 Mar 8;23:115. doi: 10.1186/s12872-023-03141-x (PMC9993504; doi:10.1186/s12872-023-03141-x)
Supplement: Supplementary file 2 — Additional file 2. Table 1. The conversion of ECHO and CMR WMSI into LVEF by regression models in 3 studies [file 12872_2023_3141_MOESM2_ESM.docx]

| Supplementary Table 1. The conversion of ECHO and CMR WMSI into LVEF by regression models in 3 studies | | | | |  |
| --- | --- | --- | --- | --- | --- |
| **WMSI** | **ECHO LVEF***  (Lebeau *et al*. N=243)(1) | **ECHO LVEF^Ϯ^**  (Moller *et al*.  N=767)(2) | **MRI LVEF^‡^**  (Lebeau *et al*.  N=122)(3) | **Approximative**  LVEF | |
| 1,0 | 67 | 64 | 64 | Normal (N) = 60% | |
| 1,1 | 65 | 62 | 62 |  | |
| 1,2 | 62 | 59 | 59 |  | |
| 1,3 | 61 | 56 | 56 |  | |
| 1,4 | 57 | 54 | 54 |  | |
| 1,5 | 54 | 51 | 51 | Mild Hypokinesia (MH) = 50% | |
| 1,6 | 53 | 48 | 48 |  | |
| 1,7 | 50 | 46 | 46 |  | |
| 1,8 | 47 | 43 | 43 |  | |
| 1,9 | 44 | 41 | 41 |  | |
| 2,0 | 41 | 38 | 38 | Hypokinesia (H) = 40% | |
| 2,1 | 39 | 35 | 35 |  | |
| 2,2 | 36 | 33 | 33 |  | |
| 2,3 | 34 | 30 | 30 |  | |
| 2,4 | 31 | 28 | 28 |  | |
| 2,5 | 28 | 25 | 25 | Severe Hypokinesia (SH)= 30% | |
| 2,6 | 26 | 22 | 22 |  | |
| 2,7 | 24 | 20 | 20 |  | |
| 2,8 | 21 | 17 | 17 |  | |
| 2,9 | 18 | 14 | 14 |  | |
| 3,0 | 15 | 12 | 12 | Akinesia (A) = 10% | |

* The linear regression predicting LVEF by WMSI used isotopic ventriculography as the reference method (LVEF = 93-26WMSI);

**^Ϯ^** A validated combination of the modified method of Quinones and an overall visual estimation on transthoracic echo was used as the reference LVEF method (regression equation: LVEF=90-26WMSI);

^‡^ LVEF can also be derived from the WMSI in CMR using the volumetric method as the reference LVEF (regression equation: LVEF=90-26WMSI)

**REFERENCES**

1. Lebeau R, Di Lorenzo M, Amyot R, Veilleux M, Lemieux R, Sauvé C. A new tool for estimating left ventricular ejection fraction derived from wall motion score index. The Canadian journal of cardiology. 2003;19(4):397-404.

2. Møller JE, Hillis GS, Oh JK, Reeder GS, Gersh BJ, Pellikka PA. Wall motion score index and ejection fraction for risk stratification after acute myocardial infarction. American heart journal. 2006;151(2):419-25.

3. Lebeau R, Serri K, Morice MC, Hovasse T, Unterseeh T, Piéchaud JF, et al. Assessment of left ventricular ejection fraction using the wall motion score index in cardiac magnetic resonance imaging. Archives of cardiovascular diseases. 2012;105(2):91-8.
