# Supplementary material for: Left ventricular ejection fraction using a simplified wall motion score based on mid-parasternal short axis and apical four-chamber views for non-cardiologists
Source: BMC Cardiovasc Disord. 2023 Mar 8;23:115. doi: 10.1186/s12872-023-03141-x (PMC9993504; doi:10.1186/s12872-023-03141-x)
Supplement: Supplementary file 7 — Additional file 7. Table 2. Correlation between a novel wall motion score method using different simplified combinations of views with the reference standard to estimate left ventricular ejection fraction [file 12872_2023_3141_MOESM7_ESM.docx]

|  | **PSAX- BMA** | **Apical 234CH** | **MID-4CH** |  |
| --- | --- | --- | --- | --- |
| **Reference echocardiographer, n=46 (r²)** | 0,87 | 0,87 | 0,90 |  |
| **Cardiologists, n=10 (r²)** | 0,89 | 0,89 | 0,84 |  |
| **Emergency Physicians, n=10 (r²)** | 0,89 | 0,89 | 0,90 |  |

Supplementary Table 2: Correlation between a novel wall motion score method using different simplified combinations of views with the reference standard to estimate left ventricular ejection fraction

PSAX-BMA: A combination of the three parasternal short-axis views (PSAX) (Mitral level base

(PSAX-B), papillary muscle level (PSAX-MID) and apex (PSAX-A));

Apical 234Ch: A combination of the three apical views (2-chambers, 3-chambers and 4-chambers);

MID-4CH: A more limited combination of PSAX-MID and apical 4-chambers view.
